# Design of amphotericin B oral formulation for antifungal therapy

**DOI:** 10.1080/10717544.2016.1225852

**Published:** 2017-02-03

**Authors:** Min Liu, Meiwan Chen, Zhiwen Yang

**Affiliations:** 1 Department of Pharmacy, Songjiang Hospital Affiliated Shanghai First People's Hospital, Shanghai Jiao Tong University, Shanghai, China,; 2 Urology Department, First Affiliated Hospital of Gannan Medical University, Gannan Medical University, Ganzhou, China, and; 3 State Key Laboratory of Quality Research in Chinese Medicine, Institute of Chinese Medical Sciences, University of Macau, Macau, China

**Keywords:** Amphotericin B, oral administration, formulation design

## Abstract

Amphotericin B (AmB) remains the “gold standard” for systemic antifungal therapy, even though new drugs are emerging as the attractive antifungal agents. Since AmB has negligible oral absorption as a consequence of its unfavorable physicochemical characterizations, its use is restricted to parenteral administration which is accompanied by severe side effects. As greater understanding of the gastrointestinal tract has developed, the advanced drug delivery systems are emerging with the potential to overcome the barriers of AmB oral delivery. Much research has demonstrated that oral AmB formulations such as lipid formulations may have beneficial therapeutic efficacy with reduced adverse effects and suitable for clinical application. Here we reviewed the different formulation strategies to enhance oral drug efficacy, and discussed the current trends and future perspectives for AmB oral administration in the treatment of antifungal infections.

## Introduction

Over the last decade, advances in health care have inadvertently led to a rapid increase in the number of patients suffering from the opportunistic fungal infections. Organ transplant recipients, cancer patients, and those with HIV/AIDS usually receive intensive care therapy that includes broad-spectrum antibiotics and/or immunosuppressants, placing them at greater risk of candidiasis, histoplasmosis, and aspergillosis invasion (Diro et al., 2015). In these patients, systemic fungal infections may account for a high mortality rate of up to more than 30%. In particular, about 90% of HIV/AIDS patients die as a result of these fungal infections (Barau et al., 2014). The treatment of systemic fungal infections in clinical practice therefore poses a major challenge (Hussain et al., 2016).

Amphotericin B (AmB) is a life-saving drug in the treatment of serious systemic fungal infections, with up to 60% effective efficacy following intravenous (IV) administration. Because of its powerful activity, AmB is still the most frequently used antifungal in hospitals despite the development of a number of new antifungal agents (Machado et al., 2015). However, AmB is not recommended as the first choice for the treatment of patients with progressive, potentially life-threatening fungal infections. This is likely because of the dose-dependent nephrotoxicity associated with the parenteral formulations of AmB (Farmakiotis et al., 2013; Messori et al., 2013). In addition to Fungizone®, lipid-based formulations for IV injection, such as Abelcet® (a lipid complex), AmBisome® (a liposomal product), and Amphocil® (a colloidal dispersion), have been developed and marketed. However, high cost and other disadvantages still limit their widespread use in clinical practice (Farmakiotis et al., 2013).

Oral administration is the most widely used route for drug delivery (Gaba et al., 2015), and is the most readily accepted form by patients. In contrast to typical IV administration, an effective and safe oral formulation of AmB could greatly reduce the medical costs, decrease the side effects, and improve patient life quality. Various carrier systems for oral delivery, including solid lipid nanoparticles (SLNs) (Chaudhari et al., 2016), PLGA nanoparticles (Italia et al., 2009), Polymer lipid hybrid nanoparticles (Jain et al., 2012), cubosomes (Yang et al., 2012), emulsions (Wasan et al., 2009; Gershkovich et al., 2010), micelles (Risovic et al., 2003), exhibited the lower nephrotoxicity compared to that of the parenteral formulation. To date, the negligible bioavailability of AmB is observed after oral administration due to poor membrane permeability, low aqueous solubility, and instability at the low pH found in the stomach (Volmer et al., 2010; Mistro et al., 2012). Following the failure of a series of clinical trials, it has been generally accepted that the oral formulations of AmB are unfeasible in clinical practice.

However, recent advances in alternative delivery routes and in-depth understanding of drug delivery technologies have an inspired reassessment of the potential for development of AmB oral formulations (Thornton & Wasan, 2009). Different research groups have developed the various carrier systems to shield AmB molecule’s unfavorable characteristics for oral delivery, including polymeric nanoparticles (Verma et al., 2011), carbon nanotubes (Benincasa et al., 2011), nanosuspensions (Golenser & Domb, 2006), polymer lipid hybrid nanoparticles (Jain et al., 2012), SLNs (Patel & Patravale, 2011), cubosomes (Yang et al., 2012), emulsions (Wasan et al., 2009; Ibrahim et al., 2012), and cochleates (Perlin, 2004). In this review, progress in the development of an oral AmB product is summarized. There are still challenges to the development of a successful oral AmB product using the current formulation technology, but lipid formulations may have better prospects for clinical application as compared to other drug delivery systems.

## Mechanism of action

Ergosterol is a critical component of fungal cell membranes, providing a target for antifungal drugs. It has been found that AmB has higher affinity for ergosterol-containing membrane than for cholesterol-containing membrane. Thus, AmB has stronger specificity and selectivity toward fungal cells compared to host cells. After AmB has bound to membrane ergosterol, a polar pore is created that changes the fungal membrane permeability. Additionally, AmB inhibits membrane enzymes such as proton ATPase, reduces proliferative ability, and depletes cellular energy reserves in fungal cells. Eventually, AmB leads to cellular dysfunction and cell death (Wilcock et al., 2013).

Toxicity of AmB arises from its weak affinity for cholesterol, the main component of the mammalian membrane. AmB interacts with cellular cholesterol component and then penetrates the mammalian membrane to create transmembrane channels that leads to the leakage of metabolites and monovalent ions triggering host cell death. As AmB molecules accumulate extensively in vital organs of the human body, particularly the kidney, its weaker affinity for cholesterol becomes deleterious to host cells via the disruption of membrane integrity, causing severe cellular damage (Neumann et al., 2010).

## Absorption mechanisms after oral delivery

### Physicochemical properties of the drugs

**Figure 1.**
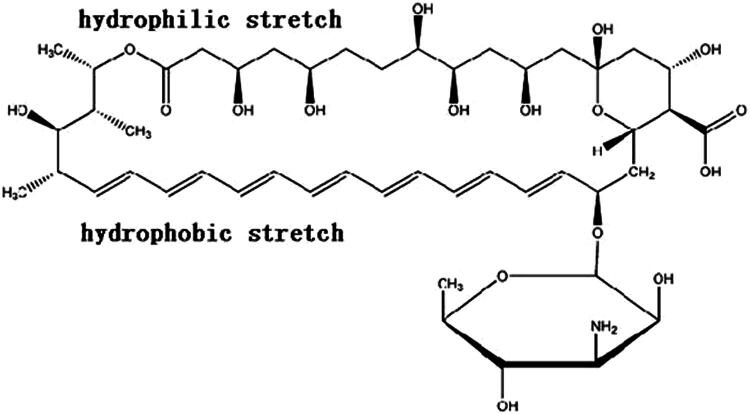
Structure of amphotericin B.

AmB is a polyene macrolide antifungal that was isolated from a strain of *Streptomyces nodosus* in 1959. AmB has a very poor solubility in the most solvents: It is almost completely insoluble in water, sparingly soluble in alcohols, but highly soluble in DMSO and DMF. The structure of AmB molecule is comprised of a macrocyclic lactone, one non-polar heptene group (Figure 1). The ring also contains a glycosylated mycosamine group at C19. An amino group in the mycosamine head group and a carboxyl group at C16 are both charged at neutral pH and determines the amphoteric nature of AmB. The three-dimensional structure of AmB with hydrophobic and hydrophilic regions further confers amphipathic properties (Shim et al., 2011). In addition, the heptene region at one end of the molecule is subject to chemical degradation at acidic pH, resulting in the carbon–carbon double bond cleavage. When the molecular weight of a drug is over 500 Da, passive absorption efficiency decreases. It is well known that AmB is mainly absorbed by passive diffusion through the intestinal membrane. The molecular formula of AmB is C_47_H_73_NO_17_ with a molecular weight of 924 Da, limiting its oral absorption. Low water solubility, poor gastrointestinal (GI) permeability, and instability in the stomach contribute to the low bioavailability of AmB by the oral route.

### Transmembrane efflux

P-glycoprotein (P-gp), a membrane-associated protein belonging to the adenosine triphosphate binding cassette (ABC) superfamily, has a dominant role in the oral bioavailability through drug efflux (Shukla et al., 2014). Peceol®, a lipid excipient, has been shown to inhibit P-gp efflux activity in a Caco-2 monolayer using the Transwell® semi-permeable cell culture support system. After treatment with Peceol® at a low concentration, P-gp protein expression is reduced in the Caco-2 cellular model (Risovic et al., 2004). At non-cytotoxic concentrations, Peceol® treatments of 0.25% (v/v) and 0.5% (v/v) reduced P-gp protein levels to 62.4% and 68.4%, respectively (Prasad et al., 2003).

To date, there is no evidence that identifies AmB as a P-gp substrate. However, incorporation of AmB into an oral formulation composed of Peceol® increased GI tract absorption (Wasan et al., 2009). After single-dose oral administration of Peceol-AmB at 50 mg/kg to rats, the maximum plasma concentrations (*C*_max_) and area under the curve (AUC_0–24 h_) were increased to 41 469 ± 891 μg/mL and 11 407 ± 4971 μg × h/mL, respectively (Risovic et al., 2007). This result indicated that there was significant enhancement in the bioavailability following oral administration. In addition, AmB was undetectable in kidney tissue following oral administration of Peceol-AmB at 50 mg/kg, indicating that incorporation of AmB into Peceol® alter AmB tissue distribution. Consequently, the Peceol-AmB formulation had a major impact on the safety, with a significant reduction in nephrotoxic potential *in vivo*. The considerable differences in tissue distribution and plasma concentrations suggested a new mechanism that may have contributed to the effect of lipid Peceol®: P-gp protein inhibition and drug transport across cell membranes occurred concurrently with Peceol® absorption from the GI tract (Risovic et al., 2003). Somewhat confusingly, a recent research reported contrary data, indicating that AmB did not stimulate P-gp activity. Consequently, AmB was not a substrate of P-gp in Caco-2 cells (Osei-Twum & Wasan, 2015).

### Lymphatic transport

The lymphatic system is part of the circulatory system, consisting of an elaborate network of lymphatic vessels throughout the body (Miteva et al., 2010; Randolph & Miller, 2014). The structure and function of the lymphatics system, which provides an alternative environment for the transport of foreign substances, are not uniform throughout the body. For example, the intestinal lymphatic system transports dietary fat and lipid soluble vitamins from the interstitium to the intravascular space. During the process of intestinal absorption, co-administered lipid stimulates lipoprotein formation to delivery drug into the bloodstream.

Peceol®, a cheap, safe, and natural lipid excipient for promoting the lymphatic transport of drugs, is associated with the triglyceride core of chylomicrons, thus increasing systemic oral absorption. To explore the lymphatic transport, the pharmacokinetics of AmB were investigated in the mesenteric and thoracic lymph following a single dose of Peceol-AmB formulation to SD rats by oral gavage. Mesenteric and thoracic lymph were collected to analyze AmB levels by high-pressure liquid chromatography method. There were significantly larger amounts of AmB in mesenteric lymph at 0–8 h intervals post-oral gavage of Peceol-AmB compared to the non-lipid group. In this study, increased systemic concentrations of AmB were attributed to access of the drug to the GI lymphatic system through intestinal epithelial cells. However, these data clearly indicated that lymphatic absorption and transport accounted for less than 10% of the total AmB concentration in rat serum. The findings suggested that incorporation of AmB into lipid formulations may address some of the oral limitations of AmB by enhancing lymphatic transport (Sachs-Barrable et al., 2008).

### Application of nanotechnology

It seems impossible to develop an oral formulation of AmB, primarily because of the physiological environment, physicochemical barriers, and the hostile acidic milieu of the stomach. Nevertheless, with the advent of drug delivery vehicles designed to overcome the low bioavailability, oral formulation after incorporation of AmB into a nanocarrier appears promising.

**Figure 2.**
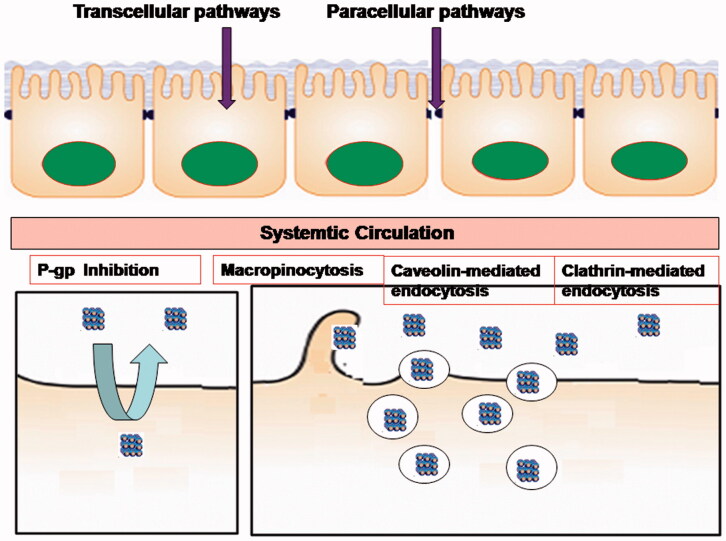
The oral absorption mechanisms of nanoparticles in intestinal tract.

A variety of nanotechnology has shown potential to enhance the oral delivery of poorly water-soluble drugs (Figure 2). Nanotechnology could address the principal disadvantages of many drugs: optimized mucoadhesion and cellular uptake; increased solubilization potential and superior encapsulation; prolonged residence time and altered absorption pathways in the GI tract; prevention of metabolic degradation within the GI tract; ability to incorporate wide variety of drug substances; and increased chemical versatility of materials eligible for nano-medicine (Fonte et al., 2015; Horev et al., 2015; Fatma et al., 2016). However, the design, selection, and development of drug delivery systems are challenging, requiring in-depth understanding of its behavior under physiological conditions and the physicochemical properties of the drug substances. Substantial efforts have been made to evaluate their *in vivo* fate of nano-drug delivery systems after oral administration (Shan et al., 2015). Particle size, shape, composition, surface properties, and function of nanoparticles play an important role in enhancing their uptake across the GI membrane. Various absorption mechanisms including enterocytes transport, mucoadhesion, tight junction modulation, receptor mediated endocytosis and transcytosis, phagocytosis via specialized microfold cells, other mucosa-associated lymphoid tissues, and lymphatic absorption were responsible for oral drug absorption. However, little has been reported on methods to increase AmB oral bioavailability. Most papers only reported their potential to enhance the oral delivery, lacking further studies on the mechanisms of AmB absorption from the different nano-formulations. It remains difficulty to select and design an appropriate drug delivery system for improving AmB oral absorption. The subsequent sections covered recent developments of a variety of nanocarriers that were designed to improve oral AmB bioavailability (Table 1).

**Table 1.** *In vivo* release profiles of AmB from various oral formulation.

Formulation	Excipient	Animal	Dose	*C*_max_ (ng/mL)	*T*_max_ (h)	AUC
Nanosuspensions	Deoxycholate sodium	–	–	–	–	–
Nanosuspensions	Tween 80/Pluronic F68	–	–	–	–	–
Nanocrystals	Tween 80/poloxamer188	–	–	–	–	–
Nanoparticles	PLGA	Rat	10 mg/kg	176.0 ± 25.8	24	7938.8 ± 1450.7AUC0–inf (ng h/mL)
Nanoparticles	GCPQ	Beagle	4 mg/kg per 2 days	250	4	–
Nanoparticles	Hitosan	–	–	–	–	–
SLN	Emulsynt® GDL/ Epikuron™ 200	Rat	200 mg/kg	124.93 ± 13.61	9	6063.311AUC_0–∞_ (ng h/mL)
Liposomes	Yolk phosphatidylcholine	–	–	–	–	–
Cubosomes	Phytantriol	Rat	10 mg/kg	316.7 ± 32.6	26.0 ± 4.9	17 002.2 ± 3400.3AUC_0 − 120_ (ng h/mL)
Cubosomes	Glyceryl monoolein	Rat	10 mg/kg	286.4 ± 35.1	25.1 ± 3.7	11 749.1 ± 2231.2AUC_0 − 120_ (ng h/mL)
Emulsion	Mono/diglyceridephospholipid	Rat	50 mg/kg	96 ± 15	12.5 ± 2.7	1534 ± 229AUC_0–24_ (ng h/mL)
Cochleate	Phosphatidylserine	Rat	10 mg/kg × 15d	51.1 ± 5.8	–	–

## Nanotechnology-based approaches for oral delivery of AmB

### Drug nanocrystals

Recently, the rapidly emerging field of drug nanocrystals has provided an alternative method to overcome the poor solubility. Drug nanocrystals are carrier-free drug nanoparticles with particle sizes ranging from 10 to 1000 nm (Wang et al., 2013). Drug nanocrystals impart greater surface area, improving the saturation solubility and enhancing the dissolution rate, and can be used for high dose drugs with the minimal amount of excipients, thereby increasing the overall bioavailability to some extent. Currently, two approaches, consisting of top-down and bottom-up methods, have been developed to obtain drug nanocrystals. Top-down approach directly reduces the raw drug size from micrometer to nanometer, while bottom-up approach dissolves drug molecules in solvent and precipitates drug nanocrystals in non-solvent.

Our group reported that a simple top-down method using magnetic stirring with very low energy input to produce AmB nanocrystals for oral administration. A surfactant mixture composed of sodium deoxycholate and carbomer was used to coat the surface of AmB nanoparticles, acting as a polymeric stabilizer to reduce the thermodynamic instability and alleviate a tendency for aggregation and sedimentation. Mastersizer laser diffraction analysis and transmission electron microscopy showed that AmB nanocrystals had a spherical shape with an average particle size of 348.9 ± 21.2 nm. The saturation solubility of AmB nanocrystals reached 6.22 ± 0.74 μg/mL, while that of unprocessed AmB was less that 10 ng/mL. About 32% of AmB from AmB nanocrystals were released in the water at 4 h, while unprocessed AmB did not reach the minimum detection limit. Saturation solubility and dissolution rate demonstrated that nanocrystals technology had the potential to improve AmB oral absorption and enhance drug concentrations in plasma (Yang et al., 2014).

In a study using a bottom-up method, liquid antisolvent precipitation technique, ethanol was selected as antisolvent and MDSO as organic solvent. AmB nanocrystals produced by this method were almost spherical with a particle size of less than 150 nm with a narrow size distribution. Equilibrium solubility of AmB nanocrystals was13-fold, and the dissolution rate 2.1-fold, greater than for the unprocessed drug, indicating a potential for improved oral absorption using the nanocrystal approach (Zu et al., 2014).

### Carbon nanotubes

Functionalization of carbon nanotubes by conjugation of AmB has emerged as an innovative and efficient tool to improve solubility, decrease toxic effects, and improve antifungal activity. Carbon nanotubes displayed broad-spectrum antifungal activity against clinical fungal strains, with minimal inhibitory concentration values ranging from 5 to 20 μg/mL, comparable to those of Fungizone®. Interestingly, AmB-conjugated carbon nanotubes exerted marked antifungal activity toward AmB resistant strains (Benincasa et al., 2011).

### Polymeric nanocarriers

AmB-loaded PLGA nanoparticles were developed to improve oral bioavailability and minimize adverse effects. The nanoparticles showed 34.5 ± 2.1% entrapment efficiency, a smooth spherical appearance, and a size of 165.6 ± 2.9 nm. An *in vitro* release study indicated an initial rapid drug release followed by sustained release in release medium containing 0.25% w/v of sodium lauryl sulfate. Furthermore, *in vivo* evaluation found detectable plasma concentrations over a period of 5 days following oral administration to rats. Relative oral bioavailability reached 800% as compared to Fungizone®, accompanied by lower hemolysis and nephrotoxicity (Italia et al., 2009). In later experiments, oral therapeutic efficacy was evaluated in mice with invasive and disseminated pulmonary aspergillosis. Treatment with 5 mg/kg AmB nanoparticles orally once daily significantly reduced fungal burden in lung and kidney, exhibiting a comparable efficacy to that of parenteral Ambisome® (Italia et al., 2011; Kumar et al., 2015).

In another study, a novel AmB formulation was administered to mice using both single-dose and multiple-dose oral administration. AmB encapsulated within GCPQ nanoparticles for the oral route displayed drug bioavailability enhancement and kidney exposure reduction. Oral particle uptake achieved specific targeted delivery to key organs, such as kidneys, liver, lung, brain, and spleen. A similar result was also confirmed in beagles. In a systemic murine model of candidiasis and aspergillosis which was regarded as the industry-standard disease models, AmB-GCPQ nanoparticles at higher oral doses showed similar antifungal efficacy to parenteral AmBisome® (Serrano et al., 2015).

Chitosan-EDTA superior microparticles were used to incorporate AmB using a simple spray-drying technology. The AmB microparticles showed higher drug loading capacity and a 12-fold enhancement of dissolution performance *in vitro*. Further, *ex vivo* permeation studies suggested enhanced transportation of AmB across the GI barrier. The findings suggested the potential for the development of an oral AmB formulation (Singh et al., 2013).

Polymer lipid hybrid nanoparticles comprised of lecithin and gelatin were used to incorporate AmB through a two-step desolvation method. The *in vivo* and *in vitro* studies clearly showed that AmB-loaded nanoparticles could increase intestinal permeability and improve oral bioavailability. Furthermore, there was significantly lower nephrotoxicity with the oral nanoparticle formulation as compared to the formulation administered parenterally (Jain et al., 2012).

### Lipid-based nanocarriers

#### Solid lipid nanoparticles

AmB was incorporated into SLNs for oral administration. In this study, AmB-loaded SLNs were stable in artificial GI fluids, showing no change in the chemical structure of AmB in acidic environment ranging from pH 1.2 to pH 6.8. After single-dose oral administration of AmB-loaded SLNs to rats, there was a clear reduction in the toxicity profile compared to the control group. Relative bioavailability and elimination half-life of AmB-loaded SLNs were significantly greater than those of plain drug, suggesting that SLNs were successful in controlling the release of AmB and promoting oral delivery of AmB (Patel & Patravale, 2011). In later experiments, relative bioavailability and *C*_max_ of AmB-loaded SLNs were increased 1.05-fold and 0.78-fold compared to those of the marketed parenteral formulation, Fungizone®. This was a very promising result indicating the oral formulation could be used to treat systemic fungal infections, achieving a better therapeutical efficacy compared to IV administration. Studies indicated very low renal toxicity when given as AmB-loaded SLNs compared to the marketed formulation Fungizone®, which was further confirmed by *in vivo* distribution studies (Chaudhari et al., 2016).

#### Liposomes

AmB liposomes formulated with vegetal ceramides or phosphatidylcholine were investigated for potential oral administration. After entrapment of AmB, liposomes containing vegetal ceramides had the better membrane stability in comparison to liposomes containing phosphatidylcholine. In addition, the drug encapsulation efficiency of liposomes containing ceramides was as high as 75%, which was similar to that with liposomes containing phosphatidylcholine. The two types of liposomes had good stability in digestive medium, with or without digestive enzyme, but vegetal ceramides liposomes appeared to have the better stability than phosphatidylcholine liposomes. These results indicated that incorporation of AmB into liposomes composed of vegetal ceramides has high potential for oral administration (Skiba-Lahiani et al., 2015).

#### Cubosomal nanoparticles

Cubosomes are formed by self-assembly of amphiphilic or surfactant-like molecules and have a unique liquid crystalline structure that protects the drug from chemical degradation in the GI tract (Mat Azmi et al., 2015). Cubosomes composed of lipid make contact with endothelial cells more easily and enhance overall bioavailability of the formulation. As previously reported by our group, phytantriol was selected to develop an oral formation of AmB-loaded cubosomes, since phytantriol-based cubosomes have improved chemical stability and can withstand digestive conditions. Our group successfully overcame the poor solubility of AmB in phytantriol (<1 mg/g) using SolEmuls® technology. AmB-loaded cubosomes were prepared with an encapsulation efficiency of 91.8% and a drug loading rate of 3.0%, providing an acceptable amount of lipid and drug for oral administration. After administration of a single oral dose at 10 mg/kg, the plasma drug profile of AmB indicated sustained release with levels approximately 2.85-fold higher than with Fungizone®. In addition, BUN and PC levels in rats did not increase, suggesting low nephrotoxicity *in vivo*. However, oral administration of AmB-loaded cubosomes at a high dose gave a higher *C*_max_ than Fungizone® (461.6 ng/mL versus 218.5 ng/mL) (Yang et al., 2012). Much effort continues to be made to facilitate oral transport of AmB.

Recently, many papers have been focused on glyceryl monoolein–lipid compositions to improve AmB absorption in the GI tract. Our group recently incorporated AmB into glyceryl monoolein cubosomes for oral administration (Yang et al., 2014). After oral administration to rats at 10 mg/kg, AmB-loaded cubosomes resulted in higher *C*_max_ and AUC values compared to the control group. Furthermore, an *in vivo* antifungal study showed that AmB-loaded cubosomes dosed for two consecutive days by the oral route could significantly reduce the fungal burden in rat kidney. Transport mechanisms of GMO cubosomes across Caco-2 cells contributed to both clathrin- and caveolae-mediated endocytosis, which facilitated an increase in the oral absorption of AmB. Since the ester-based structure of glyceryl monoolein could be cleaved by pancreatic lipase, it is expected that AmB-loaded cubosomes rapidly release drug in the stomach. Thus, further work is required to achieve a better alternative for oral delivery of AmB.

#### Cochleates

**Figure 3.**
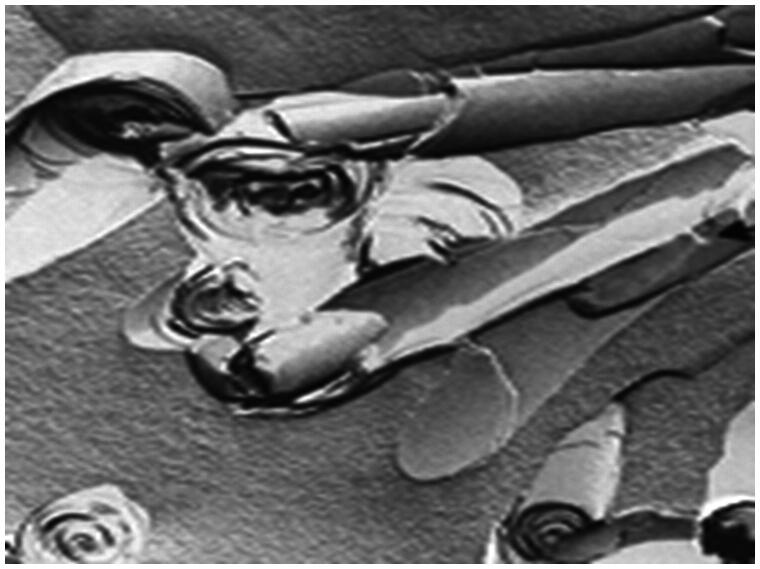
Freeze–fracture electron micrographs of oral AmB nanocochleates (Thornton & Wasan, 2009).

Cochleates (Figure 3) are novel nanostructured lipid carriers composed of negatively charged phospholipid and calcium cations (Bozo et al., 2015). After oral administration of 10 mg/kg/day for 10 days, AmB-loaded cochleates showed a significant increase in bioavailability and effective tissue accumulation in brain, liver, lung, spleen, and kidneys. In addition, with doses of up to 50 mg/kg/day in rats for 14 days, the relative toxicity of oral AmB-loaded cochleates appeared reduced compared to IV administration (Perlin, 2004). The AmB cochleate formulation was assessed for therapeutic efficacy in mouse models of aspergillosis. Administration of oral cochleates gave a dose-dependent reduction of mortality, which resulted in a better therapeutic index and a survival rate of 70% (Delmas et al., 2002). The AmB cochleate formulation was also evaluated in a disseminated candidiasis model in rats. The results indicated that 100% of the mice treated orally with cochleates survived for 16 days, while 100% mortality was observed in untreated mice by day 12. The fungal tissue burden of *Candida albicans* in kidneys and lungs was dose-dependently reduced, with a maximum 3.5-log reduction in total cell counts. In particular, complete clearance of the organism from the lungs was observed at 2.5 mg/kg/day of oral AmB cochleates (Santangelo et al., 2000). The high efficacy and low toxicity of AmB cochleates can be explained by direct fusion between the outer lipid layer of cochleates and the fungal cell membrane, but it is not known whether cochleates are absorbed and enter the circulation as intact particles (Zarif et al., 2000). In February 2009, AmB-loaded cochleates developed by the University of British Columbia (licensed to BioDelivery Sciences International, Raleigh, NC) gave a favorable outcome after oral administration in a Phase I trial, with a positive therapeutic effect equivalent to IV therapy (Thornton & Wasan, 2009). Since then, however, there have been no further reports on clinical application. In contrast, this laboratory recently developed an alternative lipid-based oral formulation of AmB, suggesting that development of AmB cochleates for oral administration was not possible.

#### Emulsions

**Figure 4.**
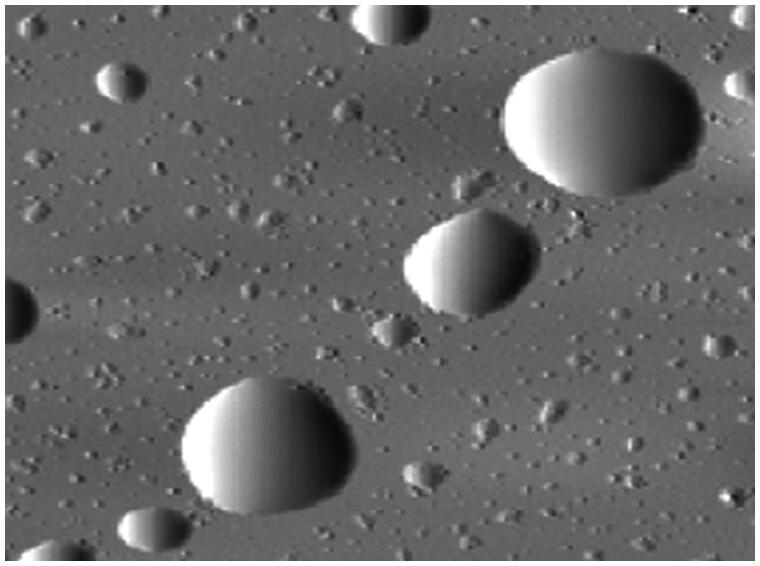
Atomic force microscopy images of oral AmB emulsion (Thornton & Wasan, 2009).

As reported previously, Peceol® excipient was approved by the FDA, and can enhance AmB aqueous solubility by 50-fold (Ibrahim et al., 2013). A simple suspension of AmB in Peceol® showed a significant increase in oral absorption and good efficacy against systemic *Aspergillius* in a rat model. Based on the preliminary experiments *in vivo and in vitro*, the University of British Columbia has successfully developed a second-generation lipid-based formulation for oral administration of AmB (Figure 4), which was licensed to iCo Therapeutics Inc., Vancouver, BC, Canada. The novel lipid-based formulation incorporated AmB into Peceol®/DSPE-PEG2000, and the obtained emulsion had significant antifungal activity and improved oral bioavailability (Gershkovich et al., 2009). First, *in vitro* experiments were used to explore the optimal parameters of the AmB formulation for bioavailability enhancement. The AmB emulsion in Peceol®/DSPE-PEG showed greater solubility of AmB (5 mg/mL) compared to the controlled group. Upon dispersion in simulated intestinal fluid, the formulation with a mean diameter of 200–400 nm was unchanged with few or no crystalline features. Drug stability in the stomach and intestine was >80% after 2 h, suggesting that AmB in Peceol®/DSPE-PEG was an excellent formulation for oral delivery. The *in vitro* cytotoxicity studies showed that AmB emulsion in Peceol®/DSPE-PEG had no cytotoxicity against monocytic (TPH1) cells while Fungizone® and AmBisome® at 500 μg/L had cytotoxicity (Leon et al., 2011). Second, *in vivo* experiments were conducted to investigate antifungal activity in a fungal-infected rat model. After oral administration at doses of 10 mg/kg to rats, AmB in Peceol®–DSPE/PEG2000 formulation facilitated intestinal diffusion and absorption into the systemic circulation with pharmacokinetic properties similar to a micellar IV preparation (Fungizone®), such as *t*_1/2_ (24.7 ± 4.8 h versus 15.2 ± 0.6 h) and AUC (1534 ± 229 h.ng/mL versus 3454 ± 198 h.ng/mL) (Gershkovich et al., 2009). Similarly to Fungizone®, orally administered AmB at 10 mg/kg dose showed homogeneous distribution in liver, spleen, lung, heart, and brain, with significant distribution toward the kidneys (Gershkovich et al., 2009). After oral treatment with Peceol®–DSPE/PEG2000-based AmB twice daily for 2 consecutive days, total fungal CFU counts recovered in all organs compared to non-treated controls, especially the kidney fungal CFU which was reduced by 95% reduction at the 10 mg/kg dose (Gershkovich et al., 2010). In addition, this formulation had little effect on the plasma creatinine levels in rats infected with *A. fumigates* and *C. albicans*, indicating that AmB incorporated into lipid-based oral formulations has negligible renal toxicity. In summer, there is growing evidence that a Peceol®–DSPE/PEG2000-based AmB formulation administered orally to rats has positive impact on the treatment of systemic fungal infections. Recently, the group from the University of British Columbia has progressed development of an oral lipid-based formulation composed of Peceol®, Gelucire 44/14, and VitE-TPGS. This formulation has showed the same high efficacy in a systemic candidiasis model in mice, and was able to increase the stability of AmB in tropical conditions (Leon et al., 2011). To overcome the instability of the above emulsion, iCo-010, a novel oral AmB emulsion, was prepared in 2015. This formulation was highly effective against murine systemic candidiasis at 43 °C for 60 days.

Based on pseudoternary phase diagrams (Capryol®90 or Capryol® PGMC as the oil phase, Tween 80 and Span 80 as the surfactant mixture), another AmB-loaded emulsion was developed as a suitable carrier for oral administration (Silva et al., 2013). The authors found time- and dose-dependent cytotoxicity in J774 cells due to a large amount of Tween and Span. They were able to improve the solubility of AmB up to 1000-fold and demonstrated coexisting monomer and aggregate phases of AmB. To our knowledge, it is difficult to determine the exact solubility in a complex system containing soluble monomers and nanoparticle aggregates. Thus, further work is required to verify the true solubility of AmB. In addition, the composition of the emulsion composition also limits its practical application because of the large amounts of Tween and Span.

#### Lipid–bile salt mixed micelles

Mixed micellar systems containing AmB and composed of bile salt and fatty acid have been reported (Dangi et al., 1998; Risovic et al., 2003). A rat gut perfusion method was used to determine membrane permeability. GI absorption of AmB was increased by more than 20-fold in the mixed micelles compared to nonmicellar and simple micellar systems. Furthermore, AmB incorporated into oral micelle formulations had less renal toxicity than the IV formulation, Fungizone®. The formulation appeared to offer a possible vehicle for oral delivery of AmB, but frequent administration and the large dose requirement limit practical treatment of fungal infections.

## Other formulations of AmB in the treatment of leishmaniasis

High pressure homogenization was used to obtain AmB nanocrystals for administration by the oral route. The obtained nanoparticles were stable inartificial GI fluids, showing no increase in particle size in acidic or neutral pH. Oral administration of AmB nanocrystals at 5 mg/kg for 4–5 consecutive days in rats significantly reduced microorganism infections in the liver compared to untreated controls, while unprocessed AmB did not show any curative effect in an *in vivo* mouse model (Kayser et al., 2003).

An oral formulation of AmB attached to carbon nanotubes was used to treat the parasite infection, *Leishmania donovani* in an established hamster model. The *in vivo* study demonstrated a 99% reduction of parasite growth in the spleen after oral administration at a dose of 15 mg/kg body weight over a 5-day course, achieving similar antileishmanial efficacy to intraperitoneal AmBisome® at 5 mg/kg (Prajapati et al., 2012).

An oral AmB emulsion resulted in a highly efficacious treatment of murine visceral leishmaniasis. Compared with the control group, oral AmB formulations at a dose of 10 mg/kg twice daily for 5 days showed a 99.5% inhibition of parasitemia in the liver. When administered intravenously at a single dose of 2 mg/kg body weight, liver parasites infection was completely eradicated. These data confirmed that an AmB oral formulation was achievable for the treatment of visceral leishmaniasis (Wasan et al., 2009).

Another oral AmB formulation, iCo-010, was developed to maintain AmB stability at relatively high temperatures ranging from 30 °C to 43 °C. Stability testing of AmB covering a 60-day period demonstrated that iCo-010 retained 90% of its original concentration. The iCo-010 formulation showed excellent anti-leishmanial activity with a 99% reduction in parasitic infection at 10 mg/kg twice daily for 5 days (Wasan et al., 2010).

An oral formulation of AmB-loaded PLGA nanoparticles was developed to evaluate the *in vitro* and *in vivo* against *Leishmania donovani.* Compared to AmB solution, the AmB nanoparticle formulation was found to clearly suppress parasite in bone marrow-derived macrophages infected with *L. donovani*. Oral AmB nanoparticles at dose from 2.5 to 20 mg/kg body weight could not suppress liver parasite burdens, indicating that the oral formulation was less effective than the IV formulation. At oral doses up to 100 mg/kg in mice, there was a significant reduction in liver parasite burdens but not in spleen or bone marrow (Italia et al., 2012).

## Future directions

Due to both the low solubility and low GI permeability of AmB, it seems impossible to develop an effective oral formulation. However, advances in the field of drug delivery have fostered new approaches for the enhancement of GI permeability and drug solubility. Over the last decade, various drug delivery vehicles have been designed to overcome the physicochemical properties exhibited by AmB. They provide better GI tract absorption and effective antifungal activity with negligible renal toxicity. However, all oral formulations are still at an early stage for the treatment of systemic fungal infections. Most of groups appear to have abandoned their research after publishing a single paper in which oral delivery vehicles have been shown to improve therapeutic efficacy. To our knowledge, most studies have remained at experimental research, lacking clinical potential. Only one group from the University of British Columbia has continued to pursue development of an oral AmB formulation over many years and published a series of papers. They have successfully developed two lipid-based formulations for oral administration. One is an oral emulsion composed of Peceol® and DSPE-PEG; the other is an oral cochleate composed of negatively charged phospholipid and calcium cations. The oral emulsion gave a favorable outcome that increased intestinal absorption of AmB, directly leading to patent acquisition by iCo Therapeutics Inc. (Vancouver, BC, Canada). The oral cochleate is currently in development by BioDelivery Sciences, Inc. (Raleigh, NC), who published results of a Phase I trial in February 2009 showing a positive therapeutic effect equivalent to IV therapy. However, it is unknown whether the two lipid-based formulations could progress to the market because further clinical data have not been reported. According to the current findings, cubosomes with a lipid composition and liquid crystalline structure have been developed to enhance the oral bioavailability of AmB, showing a better therapeutical effect on fungal infection *in vivo*. Unfortunately, there are many difficulties in the development of AmB-loaded cubosomes as a practicable oral drug delivery system. As mentioned previously, the design, selection, and development of drug delivery systems require in-depth understanding of the behavior of a drug under physiological conditions and of its physicochemical properties. However, there is still limited knowledge of how to increase AmB oral bioavailability. It remained difficult to select and design an appropriate drug delivery system to improve AmB oral absorption. Perhaps, some novel drug delivery system will emerge that can be used to develop an effective oral formulation of AmB. In spite of the difficulties, these lipid formulations may have better prospects compared to other drug delivery systems currently known. This review may provide some helpful suggestions for developing an oral formulation of AmB.

## References

[CIT0001] Barau C , Braun J , Vincent C , et al (2014). Pharmacokinetic study of raltegravir in HIV-infected patients with end-stage liver disease: the LIVERAL-ANRS 148 study. Clin Infect Dis 59:1177–84 24992955 10.1093/cid/ciu515

[CIT0002] Benincasa M , Pacor S , Wu W , et al (2011). Antifungal activity of amphotericin B conjugated to carbon nanotubes. ACS Nano 5:199–208 21141979 10.1021/nn1023522

[CIT0003] Bozo T , Brecska R , Grof P , et al (2015). Extreme resilience in cochleate nanoparticles. Langmuir 31:839–45 25521248 10.1021/la504428x

[CIT0004] Chaudhari MB , Desai PP , Patel PA , et al (2016). Solid lipid nanoparticles of amphotericin B (AmbiOnp): in vitro and in vivo assessment towards safe and effective oral treatment module. Drug Deliv Transl Res 6:354–64 26712123 10.1007/s13346-015-0267-6

[CIT0005] Dangi JS , Vyas SP , Dixit VK. (1998). Effect of various lipid-bile salt mixed micelles on the intestinal absorption of amphotericin-B in rat. Drug Dev Ind Pharm 24:631–5 9876507 10.3109/03639049809082364

[CIT0006] Delmas G , Park S , Chen ZW. T , et al (2002). Efficacy of orally delivered cochleates containing amphotericin B in a murine model of aspergillosis. Antimicrob Agents Chemother 46:2704–7 12121962 10.1128/AAC.46.8.2704-2707.2002 PMC127382

[CIT0007] Diro E , van Griensven J , Mohammed R , et al (2015). Atypical manifestations of visceral leishmaniasis in patients with HIV in north Ethiopia: a gap in guidelines for the management of opportunistic infections in resource poor settings. Lancet Infect Dis 15:122–9 25300862 10.1016/S1473-3099(14)70833-3

[CIT0008] Farmakiotis D , Tverdek FP , Kontoyiannis DP. (2013). The safety of amphotericin B lipid complex in patients with prior severe intolerance to liposomal amphotericin B. Clin Infect Dis 56:701–3 23166189 10.1093/cid/cis972

[CIT0009] Fatma S , Talegaonkar S , Iqbal Z , et al (2016). Novel flavonoid-based biodegradable nanoparticles for effective oral delivery of etoposide by P-glycoprotein modulation: an in vitro, ex vivo and in vivo investigations. Drug Deliv 23:500–11 24937381 10.3109/10717544.2014.923956

[CIT0010] Fonte P , Araujo F , Silva C , et al (2015). Polymer-based nanoparticles for oral insulin delivery: revisited approaches. Biotechnol Adv 33:1342–54 25728065 10.1016/j.biotechadv.2015.02.010

[CIT0011] Gaba B , Fazil M , Ali A , et al (2015). Nanostructured lipid (NLCs) carriers as a bioavailability enhancement tool for oral administration. Drug Deliv 22:691–700 24670099 10.3109/10717544.2014.898110

[CIT0012] Gershkovich P , Sivak O , Wasan EK , et al (2010). Biodistribution and tissue toxicity of amphotericin B in mice following multiple dose administration of a novel oral lipid-based formulation (iCo-009). J Antimicrob Chemother 65:2610–13 20861140 10.1093/jac/dkq358

[CIT0013] Gershkovich P , Wasan EK , Lin M , et al (2009). Pharmacokinetics and biodistribution of amphotericin B in rats following oral administration in a novel lipid-based formulation. J Antimicrob Chemother 64:101–8 19398459 10.1093/jac/dkp140

[CIT0014] Golenser J , Domb A. (2006). New formulations and derivatives of amphotericin B for treatment of leishmaniasis. Mini Rev Med Chem 6:153–62 16472184 10.2174/138955706775476037

[CIT0015] Horev B , Klein MI , Hwang G , et al (2015). pH-activated nanoparticles for controlled topical delivery of farnesol to disrupt oral biofilm virulence. ACS Nano 9:2390–404 25661192 10.1021/nn507170s PMC4395463

[CIT0016] Hussain A , Samad A , Singh SK , et al (2016). Nanoemulsion gel-based topical delivery of an antifungal drug: in vitro activity and in vivo evaluation. Drug Deliv 23:642–7 25013957 10.3109/10717544.2014.933284

[CIT0017] Ibrahim F , Gershkovich P , Sivak O , et al (2012). Efficacy and toxicity of a tropically stable lipid-based formulation of amphotericin B (iCo-010) in a rat model of invasive candidiasis. Int J Pharm 436:318–23 22772485 10.1016/j.ijpharm.2012.06.062

[CIT0018] Ibrahim F , Sivak O , Wasan EK , et al (2013). Efficacy of an oral and tropically stable lipid-based formulation of Amphotericin B (iCo-010) in an experimental mouse model of systemic candidiasis. Lipids Health Dis 12:158 24164705 10.1186/1476-511X-12-158 PMC4231414

[CIT0019] Italia JL , Kumar MN , Carter KC. (2012). Evaluating the potential of polyester nanoparticles for per oral delivery of amphotericin B in treating visceral leishmaniasis. J Biomed Nanotechnol 8:695–702 22852479 10.1166/jbn.2012.1414

[CIT0020] Italia JL , Sharp A , Carter KC , et al (2011). Peroral amphotericin B polymer nanoparticles lead to comparable or superior in vivo antifungal activity to that of intravenous Ambisome(R) or Fungizone. PLoS One 6:e25744 21998690 10.1371/journal.pone.0025744 PMC3188565

[CIT0021] Italia JL , Yahya MM , Singh D , et al (2009). Biodegradable nanoparticles improve oral bioavailability of amphotericin B and show reduced nephrotoxicity compared to intravenous Fungizone. Pharm Res 26:1324–31 19214716 10.1007/s11095-009-9841-2

[CIT0022] Jain S , Valvi PU , Swarnakar NK , et al (2012). Gelatin coated hybrid lipid nanoparticles for oral delivery of amphotericin B. Mol Pharm 9:2542–53 22845020 10.1021/mp300320d

[CIT0023] Kayser O , Olbrich C , Yardley V , et al (2003). Formulation of amphotericin B as nanosuspension for oral administration. Int J Pharm 254:73–5 12615413 10.1016/s0378-5173(02)00686-5

[CIT0024] Kumar R , Sahoo GC , Pandey K , et al (2015). Study the effects of PLGA-PEG encapsulated amphotericin B nanoparticle drug delivery system against Leishmania donovani. Drug Deliv 22:383–8 24601828 10.3109/10717544.2014.891271

[CIT0025] Leon CG , Lee J , Bartlett K , et al (2011). In vitro cytotoxicity of two novel oral formulations of Amphotericin B (iCo-009 and iCo-010) against *Candida albicans*, human monocytic and kidney cell lines. Lipids Health Dis 10:144 21854638 10.1186/1476-511X-10-144 PMC3173361

[CIT0026] Machado PR , Rosa ME , Guimaraes LH , et al (2015). Treatment of disseminated leishmaniasis with liposomal amphotericin B. Clin Infect Dis 61:945–9 26048961 10.1093/cid/civ416

[CIT0027] Mat Azmi ID , Wu L , Wibroe PP , et al (2015). Modulatory effect of human plasma on the internal nanostructure and size characteristics of liquid-crystalline nanocarriers. Langmuir 31:5042–9 25884233 10.1021/acs.langmuir.5b00830

[CIT0028] Messori A , Fadda V , Maratea D , et al (2013). Nephrotoxicity of different formulations of amphotericin B: summarizing evidence by network meta-analysis. Clin Infect Dis 57:1783–4 24140972 10.1093/cid/cit588

[CIT0029] Mistro S , Maciel Ide M , de Menezes RG , et al (2012). Does lipid emulsion reduce amphotericin B nephrotoxicity? A systematic review and meta-analysis. Clin Infect Dis 54:1774–7 22491505 10.1093/cid/cis290

[CIT0030] Miteva DO , Rutkowski JM , Dixon JB , et al (2010). Transmural flow modulates cell and fluid transport functions of lymphatic endothelium. Circ Res 106:920–31 20133901 10.1161/CIRCRESAHA.109.207274 PMC10994404

[CIT0031] Neumann A , Baginski M , Czub J. (2010). How do sterols determine the antifungal activity of amphotericin B? Free energy of binding between the drug and its membrane targets. J Am Chem Soc 132:18266–72 21126070 10.1021/ja1074344

[CIT0032] Osei-Twum JA , Wasan KM. (2015). Does P-glycoprotein contribute to amphotericin B epithelial transport in Caco-2 cells? Drug Dev Ind Pharm 41:1130–6 24963546 10.3109/03639045.2014.931970

[CIT0033] Patel PA , Patravale VB. (2011). AmbiOnp: solid lipid nanoparticles of amphotericin B for oral administration. J Biomed Nanotechnol 7:632–9 22195480 10.1166/jbn.2011.1332

[CIT0034] Perlin DS. (2004). Amphotericin B cochleates: a vehicle for oral delivery. Curr Opin Investig Drugs 5:198–201 15043394

[CIT0035] Prajapati VK , Awasthi K , Yadav TP , et al (2012). An oral formulation of amphotericin B attached to functionalized carbon nanotubes is an effective treatment for experimental visceral leishmaniasis. J Infect Dis 205:333–6 22158723 10.1093/infdis/jir735 PMC3244370

[CIT0036] Prasad YV , Puthli SP , Eaimtrakarn S , et al (2003). Enhanced intestinal absorption of vancomycin with Labrasol and D-alpha-tocopheryl PEG 1000 succinate in rats. Int J Pharm 250:181–90 12480284 10.1016/s0378-5173(02)00544-6

[CIT0037] Randolph GJ , Miller NE. (2014). Lymphatic transport of high-density lipoproteins and chylomicrons. J Clin Invest 124:929–35 24590278 10.1172/JCI71610 PMC3934183

[CIT0038] Risovic V , Boyd M , Choo E , et al (2003). Effects of lipid-based oral formulations on plasma and tissue amphotericin B concentrations and renal toxicity in male rats. Antimicrob Agents Chemother 47:3339–42 14506053 10.1128/AAC.47.10.3339-3342.2003 PMC201114

[CIT0039] Risovic V , Rosland M , Sivak O , et al (2007). Assessing the antifungal activity of a new oral lipid-based amphotericin B formulation following administration to rats infected with *Aspergillus fumigatus*. Drug Dev Ind Pharm 33:703–7 17654018 10.1080/03639040601077349

[CIT0040] Risovic V , Sachs-Barrable K , Boyd M , et al (2004). Potential mechanisms by which Peceol increases the gastrointestinal absorption of amphotericin B. Drug Dev Ind Pharm 30:767–74 15491054 10.1081/ddc-120039793

[CIT0041] Sachs-Barrable K , Lee SD , Wasan EK , et al (2008). Enhancing drug absorption using lipids: a case study presenting the development and pharmacological evaluation of a novel lipid-based oral amphotericin B formulation for the treatment of systemic fungal infections. Adv Drug Deliv Rev 60:692–701 18053611 10.1016/j.addr.2007.08.042

[CIT0042] Santangelo R , Paderu P , Delmas G , et al (2000). Efficacy of oral cochleate-amphotericin B in a mouse model of systemic candidiasis. Antimicrob Agents Chemother 44:2356–60 10952579 10.1128/aac.44.9.2356-2360.2000 PMC90069

[CIT0043] Serrano DR , Lalatsa A , Dea-Ayuela MA , et al (2015). Oral particle uptake and organ targeting drives the activity of amphotericin B nanoparticles. Mol Pharm 12:420–31 25558881 10.1021/mp500527x

[CIT0044] Shan W , Zhu X , Liu M , et al (2015). Overcoming the diffusion barrier of mucus and absorption barrier of epithelium by self-assembled nanoparticles for oral delivery of insulin. ACS Nano 9:2345–56 25658958 10.1021/acsnano.5b00028

[CIT0045] Shim YH , Kim YC , Lee HJ , et al (2011). Amphotericin B aggregation inhibition with novel nanoparticles prepared with poly(epsilon-caprolactone)/poly(n,n-dimethylamino-2-ethyl methacrylate) diblock copolymer. J Microbiol Biotechnol 21:28–36 21301189 10.4014/jmb.1007.07041

[CIT0046] Shukla S , Chufan EE , Singh S , et al (2014). Elucidation of the structural basis of interaction of the BCR-ABL kinase inhibitor, nilotinib (Tasigna) with the human ABC drug transporter P-glycoprotein. Leukemia 28:961–4 24418991 10.1038/leu.2014.21 PMC3981924

[CIT0047] Silva AE , Barratt G , Cheron M , et al (2013). Development of oil-in-water microemulsions for the oral delivery of amphotericin B. Int J Pharm 454:641–8 23726904 10.1016/j.ijpharm.2013.05.044

[CIT0048] Singh K , Tiwary AK , Rana V. (2013). Spray dried chitosan-EDTA superior microparticles as solid substrate for the oral delivery of amphotericin B. Int J Biol Macromol 58:310–19 23624284 10.1016/j.ijbiomac.2013.04.053

[CIT0049] Skiba-Lahiani M , Hallouard F , Mehenni L , et al (2015). Development and characterization of oral liposomes of vegetal ceramide based amphotericin B having enhanced dry solubility and solubility. Mater Sci Eng C Mater Biol Appl 48:145–9 25579907 10.1016/j.msec.2014.11.069

[CIT0050] Thornton SJ , Wasan KM. (2009). The reformulation of amphotericin B for oral administration to treat systemic fungal infections and visceral leishmaniasis. Expert Opin Drug Deliv 6:271–84 19327044 10.1517/17425240902802861

[CIT0051] Verma RK , Pandya S , Misra A. (2011). Loading and release of amphotericin-B from biodegradable poly(lactic-co-glycolic acid) nanoparticles. J Biomed Nanotechnol 7:118–20 21485832 10.1166/jbn.2011.1230

[CIT0052] Volmer AA , Szpilman AM , Carreira EM. (2010). Synthesis and biological evaluation of amphotericin B derivatives. Nat Prod Rep 27:1329–49 20556271 10.1039/b820743g

[CIT0053] Wang Y , Zheng Y , Zhang L , et al (2013). Stability of nanosuspensions in drug delivery. J Control Release 172:1126–41 23954372 10.1016/j.jconrel.2013.08.006

[CIT0054] Wasan EK , Bartlett K , Gershkovich P , et al (2009). Development and characterization of oral lipid-based amphotericin B formulations with enhanced drug solubility, stability and antifungal activity in rats infected with *Aspergillus fumigatus* or *Candida albicans*. Int J Pharm 372:76–84 19236839 10.1016/j.ijpharm.2009.01.003

[CIT0055] Wasan EK , Gershkovich P , Zhao J , et al (2010). A novel tropically stable oral amphotericin B formulation (iCo-010) exhibits efficacy against visceral leishmaniasis in a murine model. PLoS Negl Trop Dis 4:e913 21151883 10.1371/journal.pntd.0000913 PMC2998436

[CIT0056] Wasan KM , Wasan EK , Gershkovich P , et al (2009). Highly effective oral amphotericin B formulation against murine visceral leishmaniasis. J Infect Dis 200:357–60 19545212 10.1086/600105

[CIT0057] Wilcock BC , Endo MM , Uno BE , et al (2013). C2'-OH of amphotericin B plays an important role in binding the primary sterol of human cells but not yeast cells. J Am Chem Soc 135:8488–91 23718627 10.1021/ja403255s PMC3753100

[CIT0058] Yang Z , Chen M , Yang M , et al (2014). Evaluating the potential of cubosomal nanoparticles for oral delivery of amphotericin B in treating fungal infection. Int J Nanomedicine 9:327–36 24421641 10.2147/IJN.S54967 PMC3888350

[CIT0059] Yang Z , Liu M , Chen J , et al (2014). Development and characterization of amphotericin B nanosuspensions for oral administration through a simple top-down method. Curr Pharm Biotechnol 15:569–76 25005312 10.2174/1389201015666140706160709

[CIT0060] Yang Z , Tan Y , Chen M , et al (2012). Development of amphotericin B-loaded cubosomes through the SolEmuls technology for enhancing the oral bioavailability. AAPS PharmSciTech 13:1483–91 23090113 10.1208/s12249-012-9876-2 PMC3513470

[CIT0061] Zarif L , Graybill JR , Perlin D , et al (2000). Antifungal activity of amphotericin B cochleates against *Candida albicans* infection in a mouse model. Antimicrob Agents Chemother 44:1463–9 10817694 10.1128/aac.44.6.1463-1469.2000 PMC89898

[CIT0062] Zu Y , Sun W , Zhao X , et al (2014). Preparation and characterization of amorphous amphotericin B nanoparticles for oral administration through liquid antisolvent precipitation. Eur J Pharm Sci 53:109–17 24345795 10.1016/j.ejps.2013.12.005

